# Supplementary material for: DMA, a Small Molecule, Increases Median Survival and Reduces Radiation-Induced Xerostomia via the Activation of the ERK1/2 Pathway in Oral Squamous Cell Carcinoma
Source: Cancers (Basel). 2022 Oct 7;14(19):4908. doi: 10.3390/cancers14194908 (PMC9562201; doi:10.3390/cancers14194908)
Supplement: Supplementary file 1 [file cancers-14-04908-s001.zip › Final Cancer Supplemental Materials 11.06.2022.pdf]

**Supplemental Materials: DMA, a small molecule increases median survival and reduce radiation-induced xerostomia via activation of ERK1/2 pathway in Oral Squamous Cell Carcinoma**

**Palak Parashar, Monoj Kumar Das, Pragya Tripathi, Tejinder Kataria, Deepak Gupta, Deepak Sarin, Puja Panwar Hazari , and Vibha Tandon**

## Supplementary Methods

### M1. Histological studies of tissue sections of HK-1 xenograft and PDX mice models

The Crl: NU (NCr)-Foxn1$^{nu}$mice was sacrificed at the end of the study by a*sphyxiation* from *carbon dioxide* (*$CO_2$*). Organ perfusion was performed by injecting approximately 20mL of PBS into the left ventricle of the mice. The tumor and the organs such as the intestines, lungs, and spleen were collected after sacrifice, washed with PBS, soaked in blotting paper, and immediately fixed in 10% neutral formalin buffer. Formalin-fixed tissues were trimmed and dehydrated using increasing grades of alcohol (70%, 95%, and 100%), cleared with xylene, and finally infiltrated with paraffin. The 5µm tissues were sectioned using a rotary microtome (Leica RM2245) and stained with hematoxylin and eosin (H&E).

### M2. Immunohistochemistry of AQP5, Ki-67, and CD44 positive cells in HK-1 xenograft and PDX mice model

Tissue sections from the tumor, lungs, small intestine, and spleen samples (three animals/group) were evaluated for Ki-67 analysis, the salivary gland was assessed for aquaporin 5 (AQP5) expression and tumor tissue for CD44 by randomly selecting three fields per tissue section. Formalin-fixed paraffin-embedded tumor tissues were sectioned at 4–6 µm thickness using a rotary microtome (Leica RM2245), and the sections were mounted on microscopic slides. Tissue sections were deparaffinized by warming on a flattening table (Leica) at 60°C for 10 min, followed by xylene treatment. Sections were rehydrated with ethyl alcohol at gradient concentrations of 100%, 90%, and 80%, respectively, followed by washing with PBS-T washing buffer (0.3% Triton X-100 in PBS). After rehydration, antigen retrieval was performed by incubation in citrate buffer (0.01M, pH 6.0) at 90–95°C for 20 min. Tissue sections were then subjected to the blocking of endogenous peroxidase activity by incubating the sections in a 3% $H_2O_2$ solution in PBS at 25°C for 10 min. After washing with buffer for 5 min, the slides were incubated in the blocking solution (1% BSA in PBS) for 1h at 25°C. The sections were incubated for 1h in anti-Ki67 primary antibody (1:300 dilution in 1% BSA in PBS), anti-CD44 and anti-AQP5 primary antibody[1:100 dilution in tris-buffered saline with 0.1% tween 20 detergent (TBST)] solutions in a humidified chamber maintained at 25°C. After incubation, the sections were rinsed, and excess washing buffer was added. Anti-Rabbit IgG and anti-mouse IgG were used as secondary antibodies against the respective primary antibodies and added to the sections at a dilution ratio of 1:800 in PBS and incubated at 25°C for 30 min. After a series of washes with PBS, sections were incubated in IHC Select® Streptavidin-HRP for 30 min at 25°C. After another series of washes with PBS, sections were incubated in the 3, 3′-diaminobenzidine (DAB) solution for 1–2 min. After being washed with PBS (three times for 2 min each), tissue sections were counterstained by immersing them in the hematoxylin solution for <1 min. The slides were rinsed in running tap water to remove excess stain. Sections were dehydrated using 80%, 90%, and 100% ethyl alcohol. Slides were cleared in xylene three times before mounting with coverslips using an appropriate mounting medium. Microscopy was performed with Nikon TE-2000S microscope and DSFi3 camera for Ki-67, AQP5, and CD44, respectively, and NIH ImageJ was used for quantification. Ki-67, AQP5, and CD44 of each group, respectively.

### M3. Immunohistochemistry of Integrin β3 in PDX mice model

Immunohistochemistry of Integrin β3 was performed using a kit namely Poly Excel DAB detection system by PathnSitu, Biotechnologies Pvt. Ltd, USA. Tissue sections were deparaffinized in three changes of xylene. The slides were hydrated in a series of graded alcohol solutions in water. Tissue slides were blocked for 5 min with poly excel $H_2O_2$. The tissue sections were treated with a primary antibody for integrin β3 (1:100) and incubated overnight. The tissue sections were covered with a poly excel Target Binder and incubated for 10 min at 25°°C. After this, the tissue sections were

covered with poly excel polyHRP and incubated for 10 min at rt, followed by incubation in the stunn DAB solution for 5 min at room temperature. Next, the tissue sections were dipped in hematoxylin followed by a wash with distilled water. The slides were then dried and mounted with DPX and analyzed under inverted Nikon Microscope TE 2000 with a DSFI3 camera.

**M4. Hematology analysis of HK-1 xenograft mice model.**

Three mice out of each group: control, DMA (50mg/kg), radiation (4 Gy), and DMA+radiation (50mg/kg +4 Gy)were used for blood collection (100µL) from the ventricle, accessed via the left side of the chest. Blood samples were taken without an anticoagulant in a sterile 1.5 mL microcentrifuge tube for the separation of serum, which was used to measure biochemical parameters. Total serum protein (BiOLiS 24i Product No.12002022) and serum albumin levels (BiOLiS 24i Product No.12002001) were measured. The serum globulin level was calculated by subtracting the albumin level obtained from the total serum protein level. The serum aminotransferase activities of aspartate aminotransferase (AST, BiOLiS 24i Product No.12002020) and alanine aminotransferase (ALT, BiOLiS 24i Product No.12002021) were determined calorimetrically, and so were alkaline phosphatase (ALP) levels(24i Product No.12002002).

**Supplementary Figures and Table**

**Figures**

General

| | |
|---|---|
| fastp version: | 0.20.0 (https://github.com/OpenGene/fastp) |
| sequencing: | paired end (150 cycles + 150 cycles) |
| mean length before filtering: | 148bp, 147bp |
| mean length after filtering: | 147bp, 147bp |
| duplication rate: | 8.521677% |
| Insert size peak: | 239 |

Before filtering

| | |
|---|---|
| total reads: | 35.108356 M |
| total bases: | 5.193082 G |
| Q20 bases: | 5.120669 G (98.605586%) |
| Q30 bases: | 4.969532 G (95.695227%) |
| GC content: | 52.645179% |

After filtering

| | |
|---|---|
| total reads: | 35.101238 M |
| total bases: | 5.179270 G |
| Q20 bases: | 5.107252 G (98.609487%) |
| Q30 bases: | 4.956733 G (95.703304%) |
| GC content: | 52.652993% |

Filtering result

| | |
|---|---|
| reads passed filters: | 35.101238 M (99.979726%) |
| reads with low quality: | 2 (0.000006%) |
| reads with too many N: | 7.116000 K (0.020269%) |
| reads too short: | 0 (0.000000%) |

**Figure S1.** Fastp report of high quality reads of HNMT1 primary cells after removal of the adapter sequences and reads with more than 10% quality threshold (QV)<20 phred score.

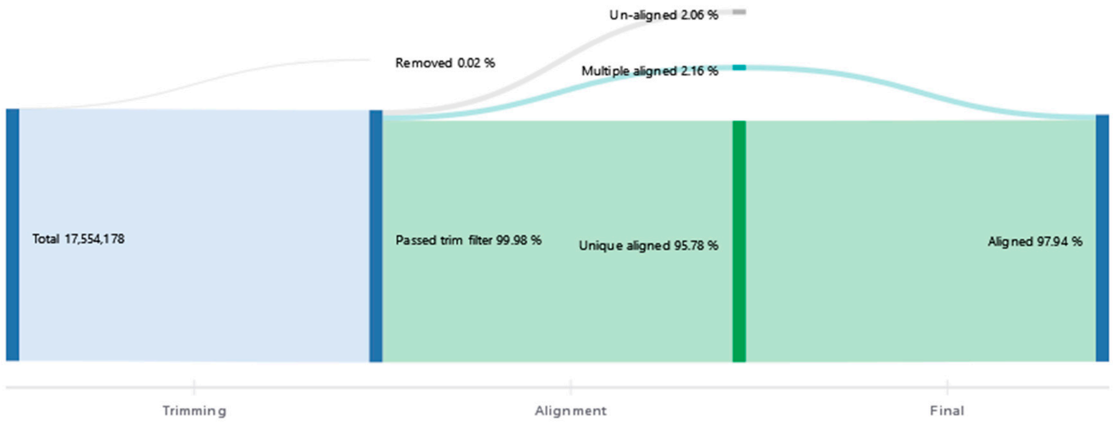


**Figure S2.** Aligned reads with the mean quality score of high quality reads of HNMT1 primary cells reads using STAR expression count.

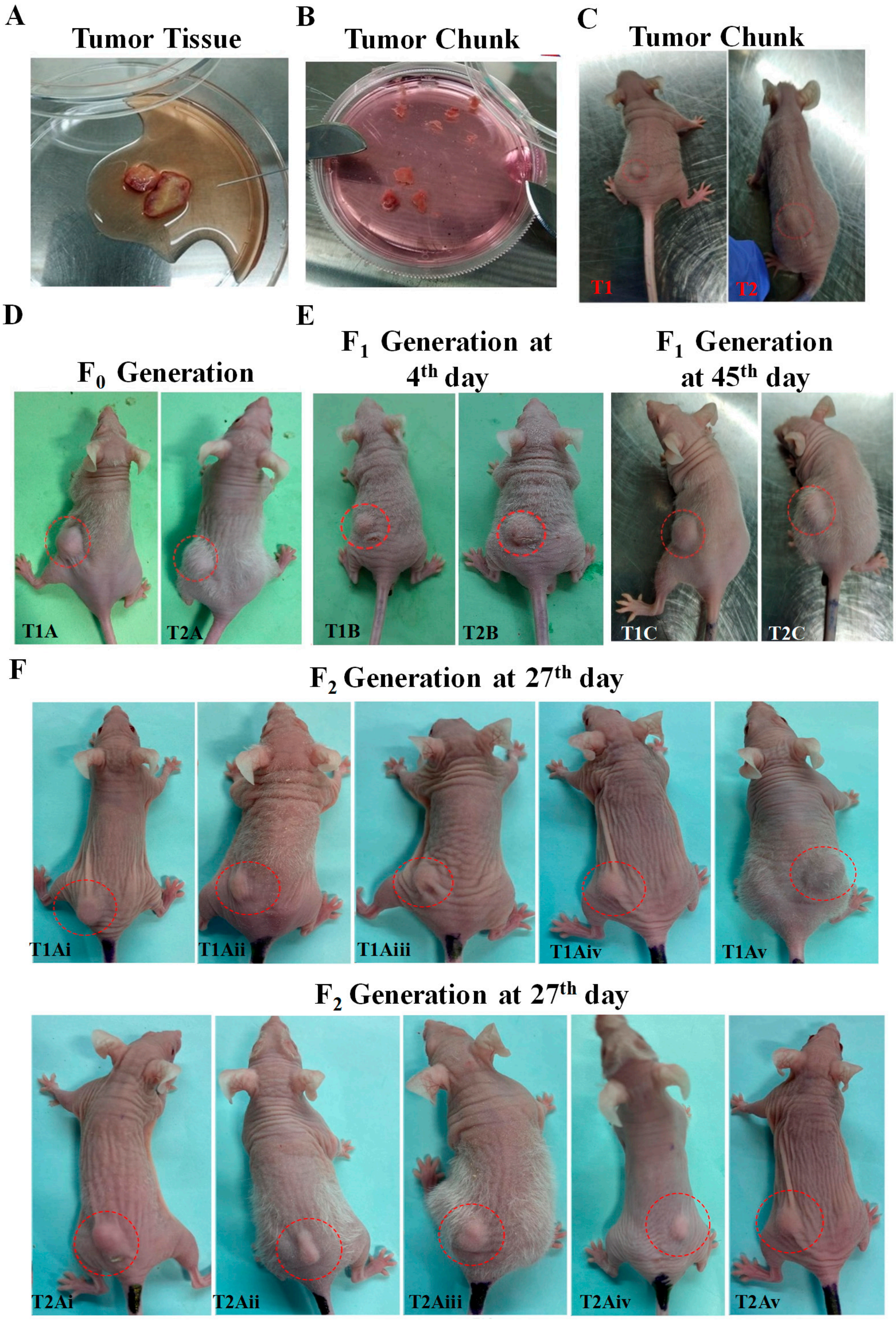


**Figure S3.** PDX mice model. Schematic outline of the development of three different generations of PDX, (**D**) $F_0$ generation, (**E**) $F_1$ generation, and (**F**) $F_2$ generation using tongue squamous carcinoma patient1 tumor implanted on the back of the nude mice

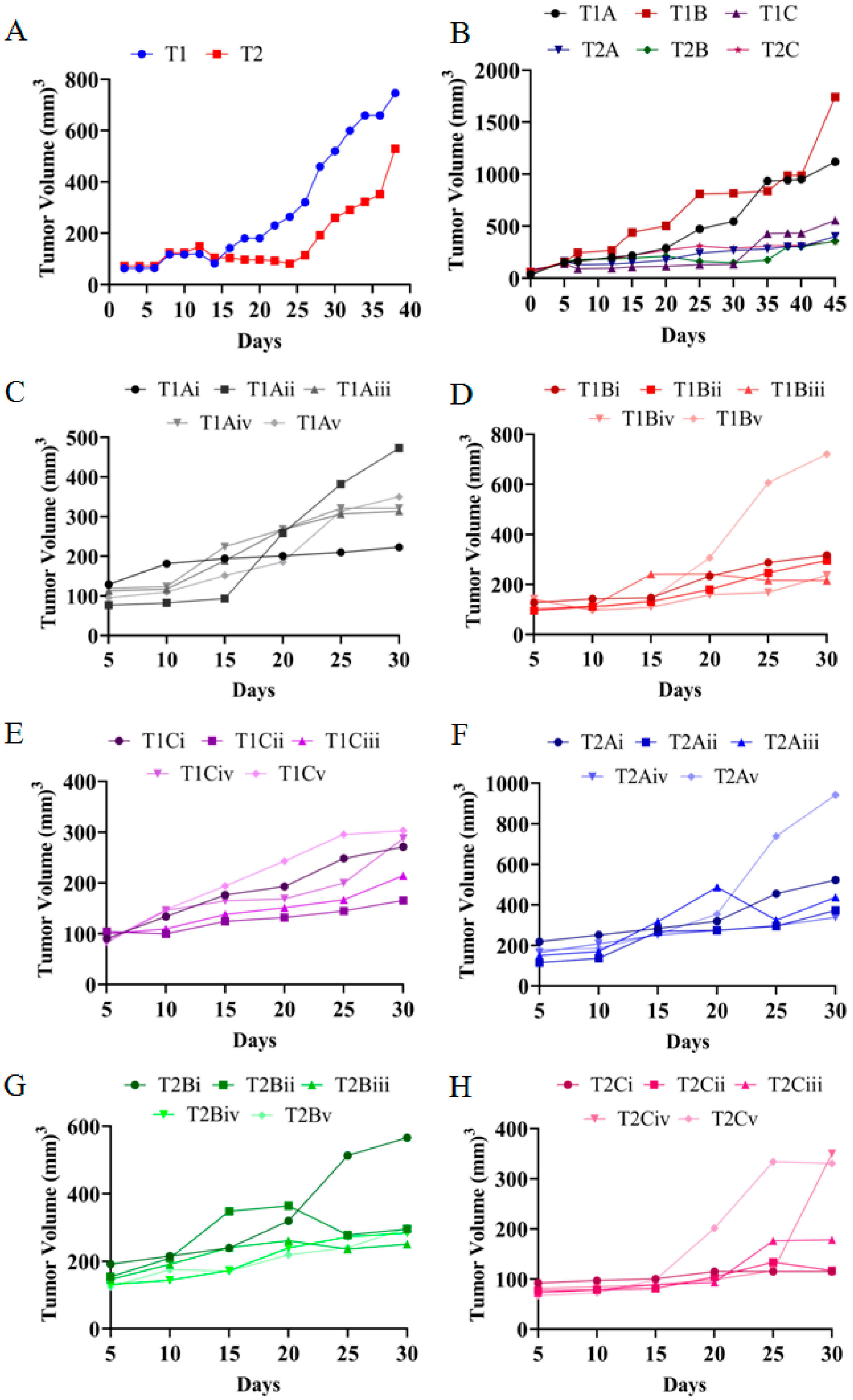


**Figure S4.** Graphical representation of the tumor formation rate of (**A**) $F_0$ generation (**B**) $F_1$ generation and (**C-H**) $F_2$ generation.

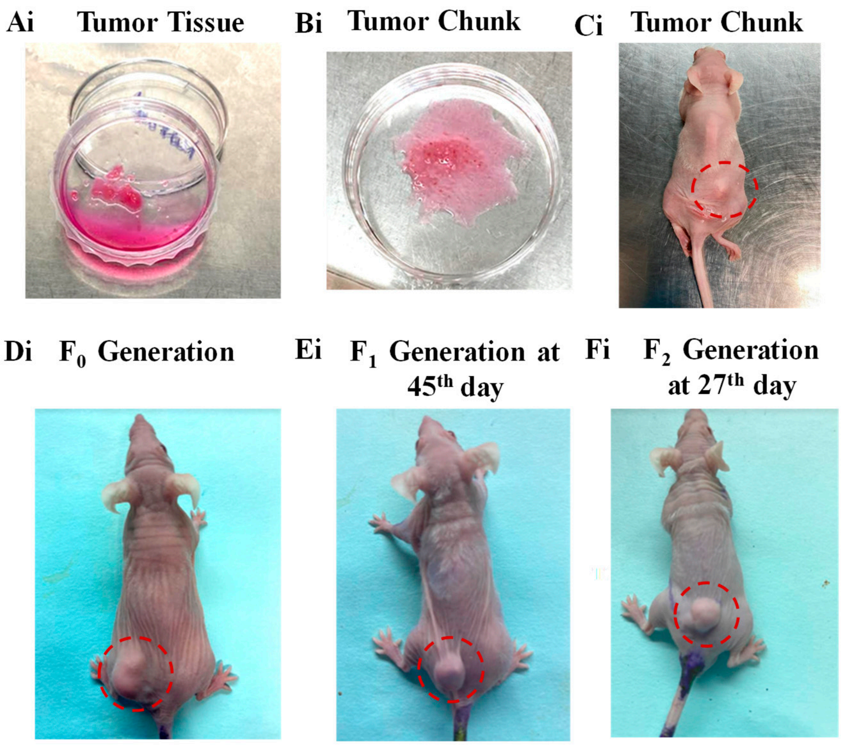


**Figure S5.** PDX mice model. Schematic outline of the development of three different generations of PDX, (**Di**) $F_0$ generation, (**Ei**) $F_1$ generation, and (**Fi**) $F_2$ generation using head and neck squamous carcinoma patient 2 tumor implanted on the back of the nude mice.

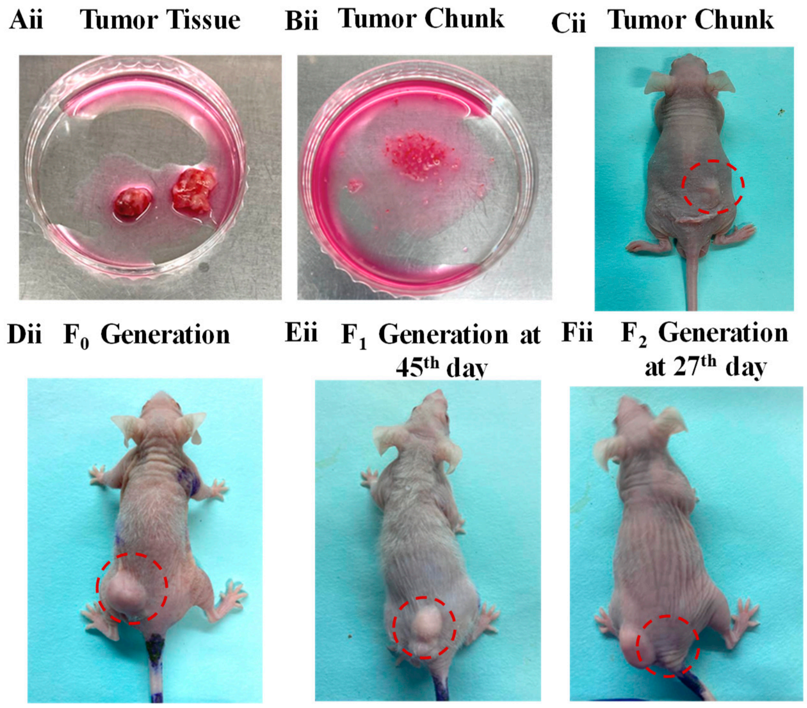


**Figure S6.** PDX mice model. Schematic outline of the development of three different generations of PDX, (**Di**) $F_0$ generation, (**Ei**) $F_1$ generation, and (**Fi**) $F_2$ generation using head and squamous carcinoma patient number 3 tumor implanted on the back of the nude mice.

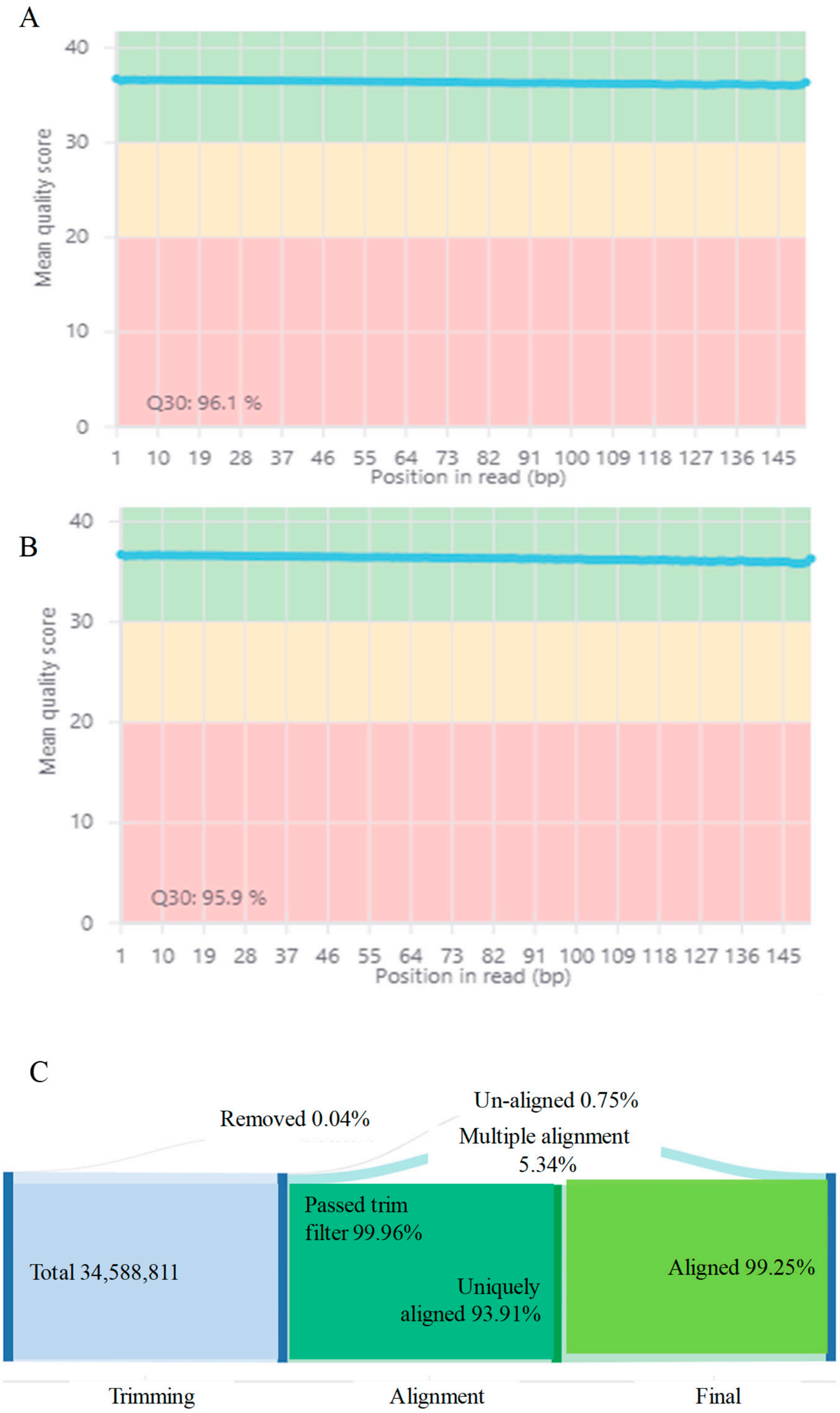


**Figure S7.**Aligned reads with the mean quality score (**A, B**) of tumor chunk 1 (T1) procured from the PDX mice model of F0 generation using STAR expression count (**C**).

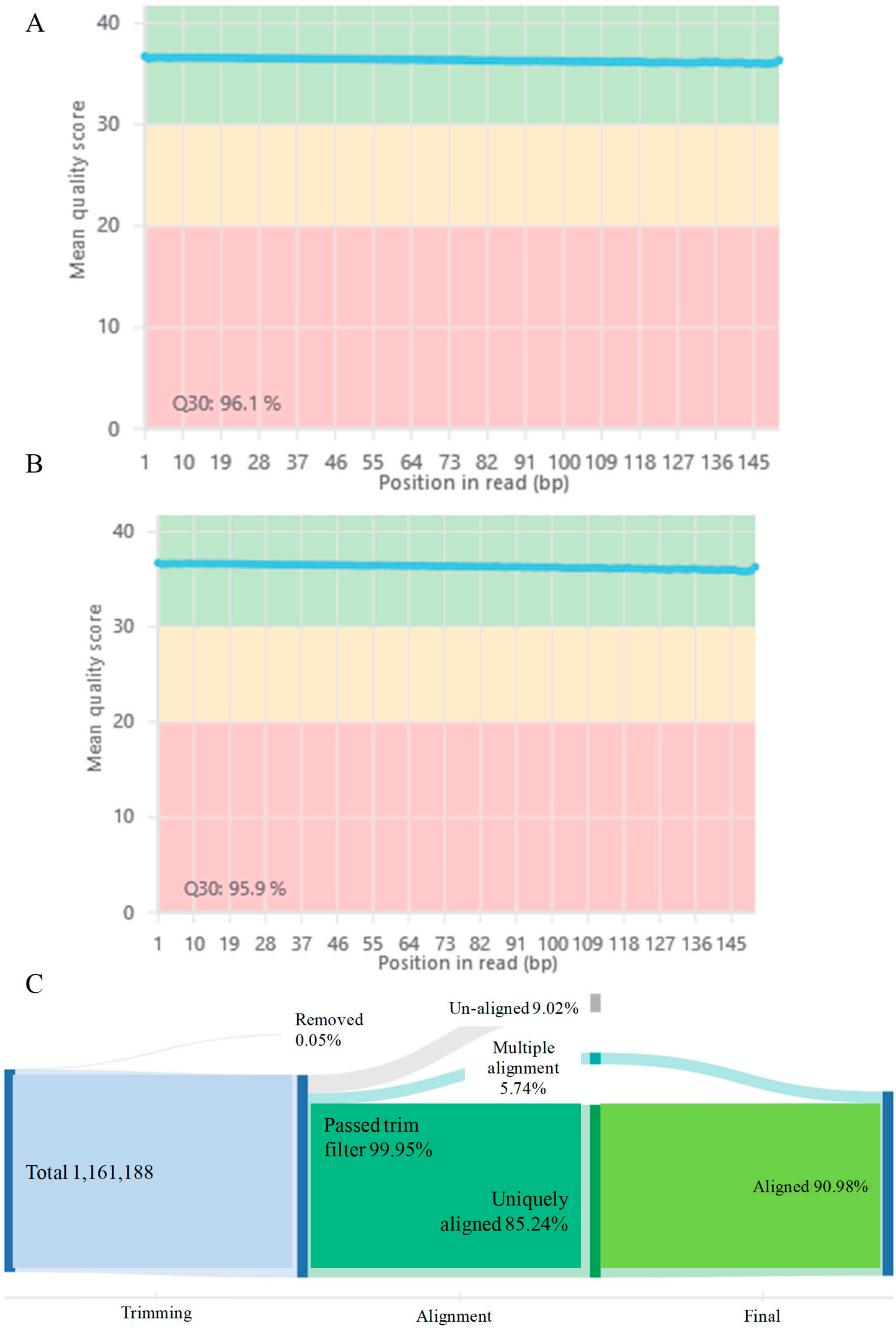


**Figure S8.** Aligned reads with the mean quality score (**A, B**) of tumor chunk 2 (T2) procured from the PDX mice model of F0 generation using STAR expression count (**C**).

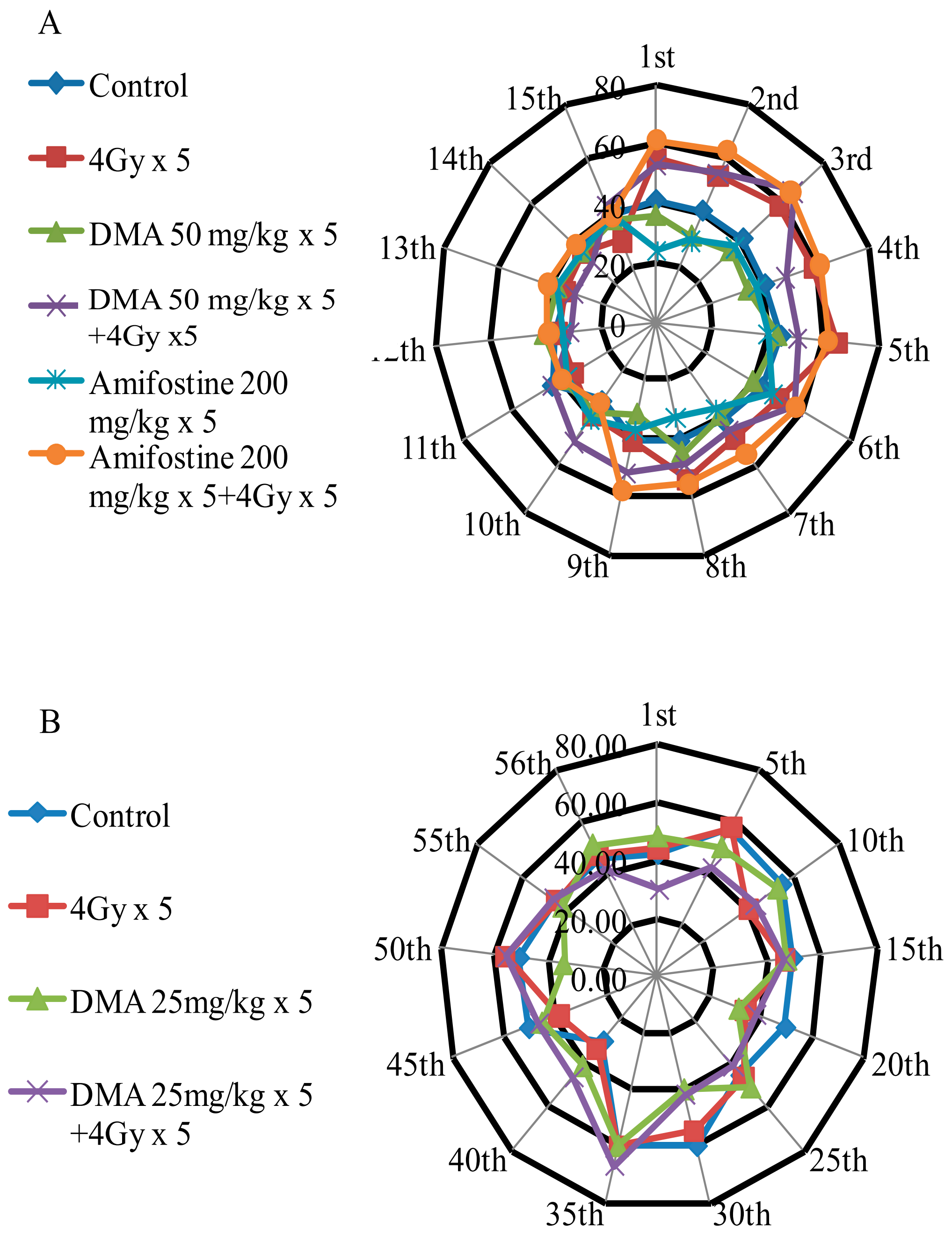


**Figure S9.** The radar plot represents the water intake (ml) in each group of tumor-bearing mice (**A**) HK-1 xenograft (**B**) PDX. Groups of 4-5 week old male and female mice were treated with DMA, focal radiation, and DMA+radiation with a dose rate of 1.2 Gy/min for consecutive 5 days.

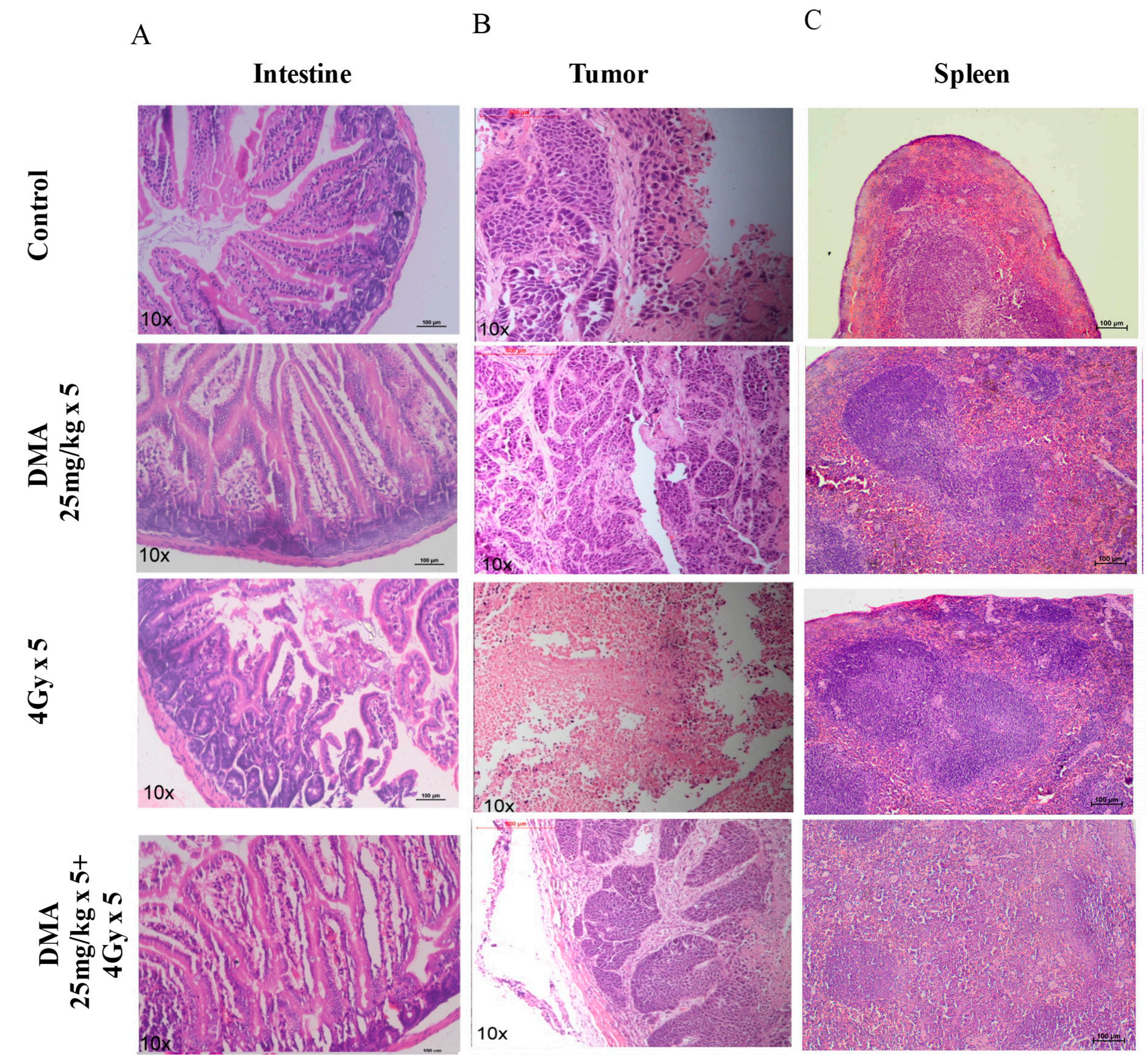


**Figure S10.** Histology of PDX tumor-bearing mice tissue sections with H&E staining on the day (60 days) of completion of the experiment. (H&E, magnification×10); Spleen, Intestine and tumor tissue of control, DMA, radiation, radiation+DMA of nude mice. Represented images were captured in Nikon 80*i* bright field microscope

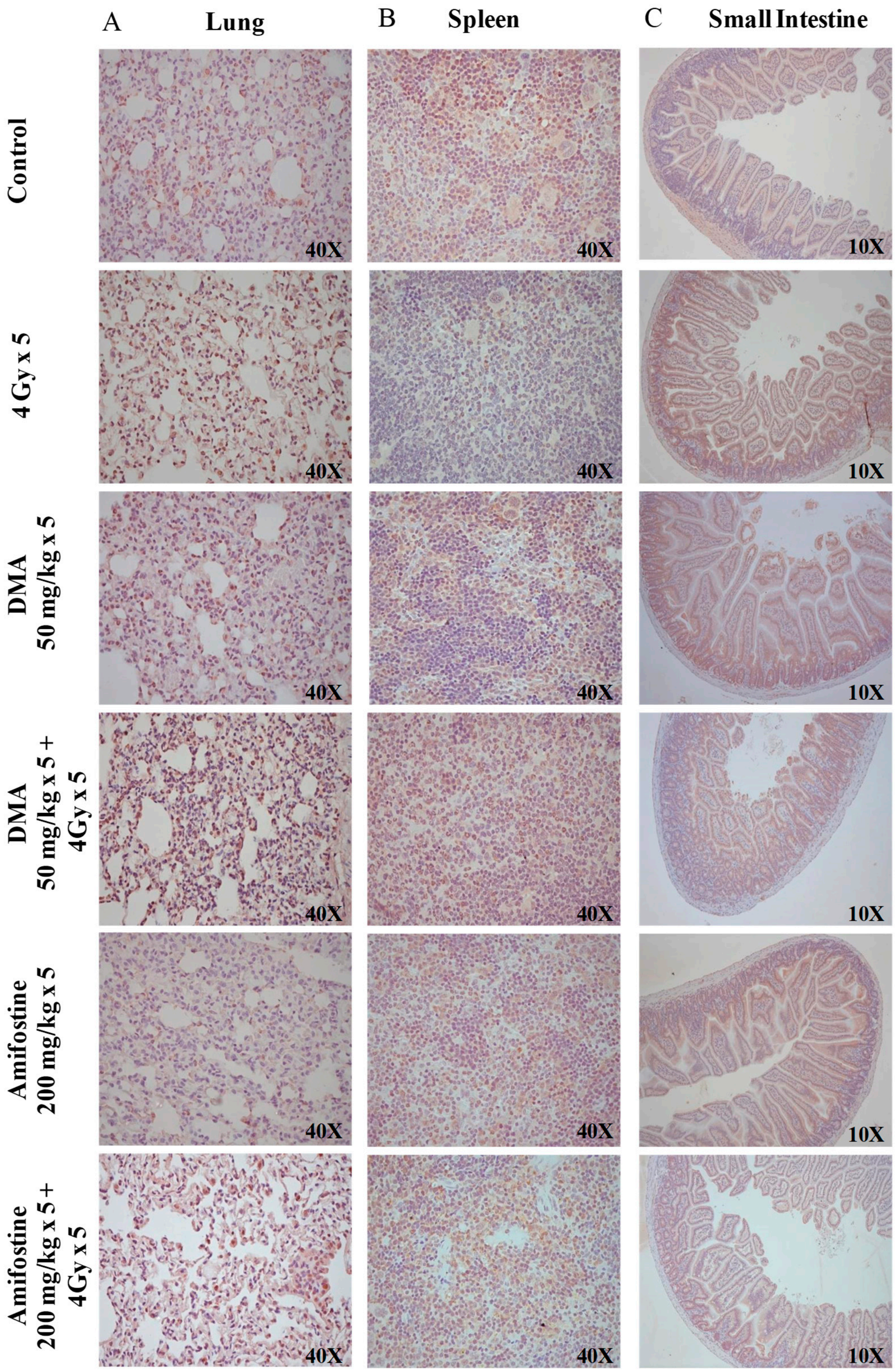


**Figure S11.** Representative images of IHC of tissue procured from HK-1 xenograft stained with Ki-67 antibody as a marker for cellular proliferation in **(A)** lung, **(B)** spleen, and **(C)** small intestine from each group and quantitation using the IHC profiler plug-in from the NIH Image J software (**Figure 6D**). The overall score is considered as the number of Ki-67 positive cells. Images were captured at 40X and 10X magnification, scale bar of 100μm.

Tables

**Table S1. List of the antibodies, software, and the reagent used in the study with their Source (Company name), Catalog number, and Unique RRID code.**

| Reagent | Source | Identifier |
|---|---|---|
| **Antibodies** | | |
| Ki-67 | Cell Signaling Technology, Inc. | Cat# 12790, RRID: AB_2631166 |
| AQP5 (D-7) | Santa Cruz Biotechnology, Inc. | Cat# sc-514022, RRID: AB_10891784 |
| CD44 | Becton Dickinson (BD) India Private limited | Cat#, RRID: AB_2229289 |
| Cleaved caspase 3 | Cell Signaling Technology, Inc. | Cat#2922S, RRID: AB_2228523 |
| Anti-Ki-67 | BioLegend | Cat# 652402, RRID:AB_11204254 |
| Anti-Ki-67 antibody | Sigma-Aldrich | Cat# AB9260, RRID: AB_2142366 |
| Ras (D2C1) Rabbit mAb | Cell Signaling Technology, Inc | Cat# 8955, RRID:AB_2797685 |
| Cyclin B1 (V152) | Cell Signaling Technology, Inc | Cat# 4135S |
| Cleaved Caspase-3 | Cell Signaling Technology, Inc | Cat#9664S |
| p44/42 MAPK | Cell Signaling Technology, Inc | Cat#4696S |
| GAPDH | Santa Cruz Biotechnology | Cat# sc-47724 |
| Anti-Rabbit IgG (whole molecule)–Biotin antibody produced in goat | Sigma-Aldrich | Cat# B8895, RRID: AB_258649 |
| Anti-Mouse IgG | Sigma -Aldrich | Cat# A9044, RRID: AB_258431 |
| Anti-Integrin β3 ITGB3 Rabbit Monoclonal Antibody | Biosource | Cat#M00587-1 |
| **Softwares** | | |
| Sigma Plot | Systat Software, Inc. | RRID:SCR_010285 |
| Image J | National Institute of Health (NIH) | RRID: SCR_003070 |
| GraphPad 8.0 | GraphPad Software, Inc. | RRID: SCR_002798 |
| **Chemicals and reagent** | | |
| AMG510 (Sotorasib) | Allianz Bioinnovation | CAS No. 2296729-00-3 |
| Dulbecco's Modified Eagle's | Sigma-Aldrich | Cat# D5523-10L |

| | | |
|---|---|---|
| Medium (DMEM) | | |
| Minimum Essential Medium Eagle (EMEM) | Sigma-Aldrich | Cat# M0643-10L |
| RPMI-1640 | Sigma-Aldrich | Cat# R6504-10L |
| McCoy's 5A | Sigma-Aldrich | Cat# M4892-10L |
| Fetal Bovine Serum (FBS) | ThermoFisher Scientific-US | Cat# 10270-106 |
| 1X antibiotic, antimycotic solution | Sigma-Aldrich | Cat# A5955-100ml |
| MTT | Sigma-Aldrich | Cat# M2128-1G |
| FITC Annexin V Apoptosis Detection Kit I | Becton Dickinson (BD) India Private limited | Cat# 556547 |
| PolyExcel HRP/DAB Detection System | PathnSitu Biotechnologies Pvt Ltd | Cat# OSH001 |
| Trypsin | Sigma-Aldrich | Cat# T4799-5G |
| Bradford | Sigma-Aldrich | Cat# B6916-500ML |
| DMSO | Merck-Life Science | Cat# 1.07046.0521 |
| DNase | HiMedia Laboratories Pvt. Ltd. | Cat# ML068 |
| Vetbond 3M adhesive | 3M | Cat# 1469SB |
| Antifade reagent | ThermoFisher Scientific-US | Cat# PM10144 |
| Matrigel Matrix | Corning Inc. | Cat# 354234 |
| Citric acid | Rankem | C2859 |
| DPX mountant liquid | HiMedia Laboratories Pvt. Ltd. | GRM655 |
| Ethyl Alcohol | CSHS fine chemicals | 64-17-5 |
| Hematoxylin (Harris) | HiMedia Laboratories Pvt. Ltd. | S034 |
| HEPES | Sigma-Aldrich | H3375 |
| Hydrogen peroxide | AVRA Synthesis Pvt. Ltd | ASH1579 |
| IHC select Streptavidin-HRP pre-diluted | Sigma-Aldrich | 20774 |
| L-Glutamine | Sigma-Aldrich | 59202C |
| D-Glucose | Merck-Life Science | CAS# 50-99-7 |

| | | |
|---|---|---|
| Normal saline | Infutec | OC90003 |
| Pilocarpine | Sigma-Aldrich | P6503 |
| Sodium Bicarbonate | Sigma-Aldrich | S8875 |
| Sodium chloride | Sigma-Aldrich | S5886 |
| Triton X-100 | Sigma-Aldrich | X100 |
| Trypan Blue Stain | Sigma-Aldrich | T8154 |
| Xylene | Rankem | X0092 |
| 3,3′-Diaminobenzidine tetrahydrochloride hydrate (DAB) | Sigma-Aldrich | D5637 |
| Potassium chloride | Merck-Life Science | CAS# 7447-40-7 |
| Disodium hydrogen phosphate | Merck-Life Science | CAS# 7558-79-4 |
| Potassium dihydrogen phosphate | Merck-Life Science | CAS# 7778-77-0 |
| EGTA | BioBasic Inc. | D0077 |
| Collagenase | Roche | 10103578001 |
| HBSS | Gibco | 13150-016 |
| Polyvinyl alcohol | Sigma-Aldrich | 341584-25G |
| Albumin Bovine fraction V (Bovine Serum Albumin, BSA) | Sisco Research Laboratories Pvt. Ltd. | CAS# 83803 |
| 40μm Nylon Cell Strainer | Corning | 352340 |

**Table S2: Incidence Scoring of Radiation Induced Histopathological Changes in HK-1 Xenograft**

| Organ | HP findings | Control | 4Gy x 5 | DMA 50 mg/kg x 5 | DMA 50 mg/kg x 5 +4Gy x 5 | Amifostine 200 mg/kg x 5 | Amifostine 200 mg/kg x 5+ 4Gy x 5 |
|---|---|---|---|---|---|---|---|
| Spleen | Extra-medullary Hematopoiesis Increased / Decreased | 0 | 4+ | 0 | 2+ | 0 | 3+ |
| Lung | Degeneration, Bronchiolar | 0 | 3+ | 0 | 4+ | 0 | 4+ |
| Tumour | Degeneration/ Necrosis, Neoplastic cells | 0 | 5+ | 0 | 5+ | 0 | 5+ |

Lesion with percent incidence: 0: No incidence; 1+: <10, 2+: 11-25, 3+: 26-50, 4+: 51-75, 5+: >75

NOTE: Tabulated scoring of the histological analysis. Control means i.v. administration of saline to the group of mice for five consecutive days, focal radiation of 4 Gy @ per day single dose x 5 = 20 Gy (total dose) through X ray irradiator with a dose rate of 1.2Gy/min.DMA 50mg/kg @ per day single i.v. dose x 5= 250 mg/kg (total dose). DMA 50 mg/kg @ per day single i.v. dose 2h before focal irradiation x 5 and 4Gy focal radiation was given for 5 days=250 mg/kg with 20 Gy (total dose).Amifostine 200mg/kg @ per day single i.v. dose = 1000 mg/kg (total dose).Amifostine 200mg/kg @ per day single i.v. dose 2h before irradiation x 5 and 4Gy focal radiation was given for 5 days = 1000 mg/kg with 20 Gy (total dose).

**Table S3: Incidence Scoring of Radiation-Induced Histopathological Changes in PDX**

| Organ | HP findings | Control | 4Gy x 5 | DMA 25 mg/kg x 5 | DMA 25 mg/kg x 5 +4Gy x 5 |
|---|---|---|---|---|---|
| Spleen | Extra-medullary Hematopoiesis Increased / Decreased | 3+ | 5+ | 3+ | 0 |
| Small Intestine | Amyloidosis, Autolysis | 0 | 4+ | 3+ | 2+ |
| Tumour | Degeneration/ Necrosis, Neoplastic cells | 0 | 4+ | 0 | 5+ |

Note: Lesion with percent incidence: 0: No incidence; 1+: <10, 2+: 11-25, 3+: 26-50, 4+: 51-75, 5+: >75

**Table S4. Liver and Kidney Function Tests in HK-1 Xenograft***

| Parameters | Normal | Control | R (4Gy x5d)[ζ] | DMA50mg/kg[†] | DMA 50 mg/kg + 4Gy[#] | Amifostine, 200 mg/kg[x] | Amifostine, 200 mg/kg + 4Gy[ϒ] |
|---|---|---|---|---|---|---|---|
| **LFT Parameters** | | | | | | | |
| Bilirubin Total (mg/dl) | 0-0.9 | 0.25 | 0.12 | 0.10 | 0.12 | 0.08 | 0.09 |
| Bilirubin Direct (mg/dl) | | 0.14 | 0.01 | 0.03 | 0.03 | 0.05 | 0.02 |
| Bilirubin Indirect (mg/dl) | | 0.11 | 0.11 | 0.07 | 0.09 | 0.03 | 0.07 |
| SGOT(AST) U/L | 54-298 | 946.5 | 292.33 | 604.74 | 541.74 | 768 | 810 |
| SGPT(ALT) U/L | 17-77 | 82.2 | 23.83 | 55 | 45.43 | 38.6 | 45.5 |
| Alkaline phosphatase U/L | 35-96 | 131.6 | 156.66 | 94 | 83.35 | 128 | 150 |
| Total Protein (g/dl) | 3.5-7.2 | 7.15 | 6.8 | 5.12 | 6.37 | 6.8 | 7.3 |
| Albumin (g/dl) | 2.5-3.0 | 3.45 | 3.03 | 3.0 | 2.88 | 2.4 | 3.1 |
| Globulin (g/dl) | 1.0-4.2 | 3.7 | 3.76 | 2.12 | 3.48 | 4.2 | 4.2 |
| A/G ratio | | 0.94 | 0.8 | 1.415 | 0.84 | 0.57 | 0.74 |
| **KFT Parameters** | | | | | | | |
| Blood Urea (mg/dl) | 17.1-70.6 | 88.5 | 59 | 45.31 | 49.31 | 54 | 64 |
| Creatinine (mg/dl) | 0.2-0.9 | 0.38 | 0.44 | 0.72 | 0.54 | 0.34 | 0.29 |
| Uric acid (mg/dl) | 1.2-7.5 | 3.32 | 2.16 | 3.15 | 2.78 | 4.2 | 3.88 |
| Calcium (mg/dl) | 7.1-10.1 | 8.7 | 9.56 | 8.2 | 9.96 | 9.0 | 9.5 |

| | | | | | | | |
|---|---|---|---|---|---|---|---|
| Phosphorous (mg/dl) | 5.7-9.2 | 7.55 | 6.8 | 6.32 | 7.42 | 8.1 | 6.7 |
| Sodium (mEq/l) | 140-160 | 146.1 | 148.36 | 152.56 | 147.56 | 150.3 | 149.3 |
| Potassium (mEq/l) | 5.0-7.5 | 5.8 | 6.16 | 6.76 | 5.77 | 5.6 | 7 |
| Chloride (mEq/l) | 88-110 | 101 | 102.33 | 92 | 103.25 | 89 | 104 |

NOTE:*Blood for liver function and kidney function test was collected without anticoagulant in 1.5 ml micro centrifuge tube, kept for 15 mins on ice after the completion of the experiment (30 days) and centrifuged at 1,4000rpm for 15 mins. ˜Control indicates administration of saline to the group of mice, ϛR =radiation through X ray with a dose rate of 1.2Gy/min, 4Gy radiation was given for 5 days; 4 Gy @ per day single dose x 5 = 20 Gy (total dose). † DMA 50mg/kg @ per day single i.v. x5 =250 mg/kg(total dose). # DMA 50mg/kg @ per day single i.v. dose 2h before irradiation x 5 and radiation 4Gy radiation was given for 5 days; 4 Gy @ per day single dose x 5 = 250 mg/kg with 20 Gy (total dose). ˣAmifostine 200mg/kg @ per day single i.v. dose x 5= 1,000 mg/kg (total dose). ˠ Amifostine 200mg/kg @ per day single i.v. dose 2h before irradiation x 5 and radiation 4Gy radiation was given for 5 days; 4 Gy @ per day single dose x 5) = 1.000 mg/kg with 20 Gy (total dose) ALT, Alanine aminotransferase; AST, Aspartate aminotransferase.
